# Mediterranean Diet and Sleep Features: A Systematic Review of Current Evidence

**DOI:** 10.3390/nu16020282

**Published:** 2024-01-17

**Authors:** Justyna Godos, Raffaele Ferri, Giuseppe Lanza, Filippo Caraci, Angel Olider Rojas Vistorte, Vanessa Yelamos Torres, Giuseppe Grosso, Sabrina Castellano

**Affiliations:** 1 Department of Biomedical and Biotechnological Sciences, University of Catania, 95123 Catania, Italy; justyna.godos@gmail.com; 2 Sleep Research Centre, Oasi Research Institute-IRCCS, 94018 Troina, Italy; rferri@oasi.en.it; 3 Department of Surgery and Medical-Surgical Specialties, University of Catania, 95123 Catania, Italy; giuseppe.lanza1@unict.it; 4 Clinical Neurophysiology Research Unit, Oasi Research Institute-IRCCS, 94018 Troina, Italy; 5 Department of Drug and Health Sciences, University of Catania, 95125 Catania, Italy; fcaraci@unict.it; 6 Neuropharmacology and Translational Neurosciences Research Unit, Oasi Research Institute-IRCCS, 94018 Troina, Italy; 7 Research Group on Food, Nutritional Biochemistry and Health, Universidad Europea del Atlántico, Isabel Torres 21, 39011 Santander, Spain; angel.rojas@uneatlantico.es (A.O.R.V.); vanessa.yelamos@uneatlantico.es (V.Y.T.); 8 Universidad Internacional Iberoamericana, Arecibo, PR 00613, USA; 9 Universidade Internacional do Cuanza, Cuito EN250, Angola; 10 Universidad Internacional Iberoamericana, Campeche 24560, Mexico; 11 Universidad de La Romana, La Romana 22000, Dominican Republic; 12 Center for Human Nutrition and Mediterranean Foods (NUTREA), University of Catania, 95123 Catania, Italy; 13 Department of Educational Sciences, University of Catania, 95124 Catania, Italy; sabrina.castellano@unict.it

**Keywords:** Mediterranean diet, sleep, insomnia, daytime sleepiness, observational studies

## Abstract

The prevalence of sleep disorders, characterized by issues with quality, timing, and sleep duration is increasing globally. Among modifiable risk factors, diet quality has been suggested to influence sleep features. The Mediterranean diet is considered a landmark dietary pattern in terms of quality and effects on human health. However, dietary habits characterized by this cultural heritage should also be considered in the context of overall lifestyle behaviors, including sleep habits. This study aimed to systematically revise the literature relating to adherence to the Mediterranean diet and sleep features in observational studies. The systematic review comprised 23 reports describing the relation between adherence to the Mediterranean diet and different sleep features, including sleep quality, sleep duration, daytime sleepiness, and insomnia symptoms. The majority of the included studies were conducted in the Mediterranean basin and reported a significant association between a higher adherence to the Mediterranean diet and a lower likelihood of having poor sleep quality, inadequate sleep duration, excessive daytime sleepiness or symptoms of insomnia. Interestingly, additional studies conducted outside the Mediterranean basin showed a relationship between the adoption of a Mediterranean-type diet and sleep quality, suggesting that biological mechanisms sustaining such an association may exist. In conclusion, current evidence suggests a relationship between adhering to the Mediterranean diet and overall sleep quality and different sleep parameters. The plausible bidirectional association should be further investigated to understand whether the promotion of a healthy diet could be used as a tool to improve sleep quality.

## 1. Introduction

The Mediterranean diet has been widely investigated as a healthy and environmentally sustainable dietary pattern, alongside being a palatable diet and relatively easy to accept in non-Mediterranean cultures [1,2]. The main features characterizing this dietary pattern include a prevalence of plant-derived foods, such as vegetables, fruit, and whole grains as the main sources of energy, vitamins, phytochemicals, and fiber; nuts and legumes as source of healthy fats and proteins; moderate intake of animal-derived foods, including meat, fish, dairy, eggs, and poultry, to be consumed alternatively; occasional consumption of elaborated sweets; adoption of extra-virgin olive oil as dressing; and moderate consumption of wine (generally occurring over the course of meals) providing peculiar phytochemicals, such as (poly)phenols [3,4]. Convincing evidence suggests that adherence to the Mediterranean diet reduces the risk of cardio-metabolic disorders, neurodegenerative diseases, and certain cancers [5]. Interestingly, several studies also investigated the potential relationship between adherence to the Mediterranean diet and other lifestyle factors potentially affecting mental and brain health [5]. Considering that the Mediterranean diet, especially when evaluated in individuals living in the Mediterranean context, should refer not only to eating habits but also to an overall lifestyle, researchers have hypothesized that adoption of such a dietary pattern could be associated with better sleep features [6].

Sleep is considered a key physiological process for the human body. Aside from its effects on the brain [7], poor sleep is also related to a higher risk of type-2 diabetes and cardiovascular disease [8]. Importantly, sleep disorders may represent a prodromic factor for mental and brain health issues, being associated with cognitive and affective disorders, as well as neurodegenerative conditions in general [9]. Lifestyle factors that may alter the circadian rhythm, such as extreme mental stress, may also contribute to the burden of sleep disorders [10]. Among other factors, dietary habits may play some role in sleep quality [6]. In this context, the joint effect of a healthy diet (such as the Mediterranean diet) and good sleep quality might be of paramount importance in preventing a large set of chronic non-communicable diseases, especially those concerning the central nervous system [11]. The aim of this study was to systematically review published studies so far exploring the relationship between adherence to the Mediterranean dietary pattern and sleep features (including quality and duration) in observational studies.

## 2. Materials and Methods

### 2.1. Study Selection

A systematic search of electronic databases (PubMed/EMBASE), from their inception up to January 2023, was conducted to identify studies examining the association between adherence to the Mediterranean dietary pattern and sleep features. Relevant terms were included in the search strategy to identify potentially relevant studies as follows: (Mediterranean diet OR dietary pattern OR dietary score OR dietary adherence) and (sleep OR apnea OR insomnia OR sleepiness) with no restrictions. Furthermore, the studies to be included were also checked to identify whether they were potentially relevant for the purpose of the present systematic review. The search, study selection, and reporting were independently performed by two authors following the Meta-analyses Of Observational Studies in Epidemiology (MOOSE) guidelines [12]. The study protocol has been registered in PROSPERO (CRD42022345280).

### 2.2. Inclusion and Exclusion Criteria

The following inclusion criteria were considered when assessing the potentially relevant studies: (i) having an observational design (such as, cohort, cross-sectional, or case-control), (ii) included adult population (≥18 years old), (iii) reported data regarding the association between the Mediterranean diet and sleep features (i.e., sleep quality, duration, daytime sleepiness, etc.). On the contrary, studies conducted on (i) children or adolescent population (<18 years old), (ii) pregnant women, and (iii) patients with end-stage degenerative diseases were not considered suitable for the purposes of this review.

### 2.3. Data Extraction

A standardized electronic form was used as guidance to extract the data from all eligible studies. The data collected were the following: (i) name of the first author and publication year; (ii) study design and location; (iii) age and sex of individuals included; (iv) sample size; (v) methods of assessment of dietary habits and adherence to the Mediterranean diet; (vi) methods of assessment of sleep-related outcomes; (vii) and summary of the study findings.

## 3. Results

### 3.1. Main Characteristics of the Included Studies

**Figure 1:**
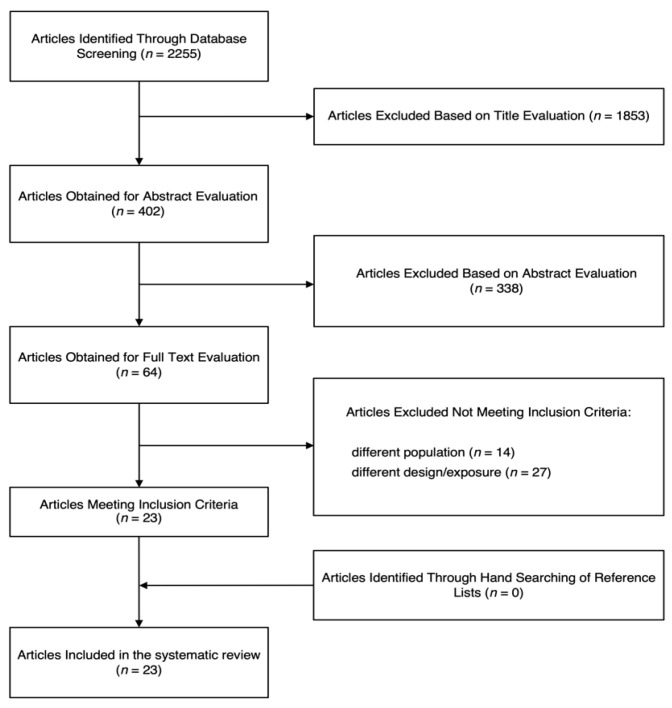
Flow chart of the study selection process.

The process of study search and selection is shown in Figure 1. Out of the initial 2255 potential articles identified, 1853 and 338 studies were not included based on the title and abstract evaluation, respectively, leaving 64 articles to be evaluated in full length. After a detailed review of the studies, 41 reports were excluded for at least one of the following reasons: 13 studies were conducted on children and/or adolescents, 27 studies did not report data on the sleep features or reported different exposure, and 1 was conducted on pregnant women. After checking for potential missing reports from the bibliographic references of the identified studies, a final pool of 23 articles [13,14,15,16,17,18,19,20,21,22,23,24,25,26,27,28,29,30,31,32,33,34,35] was selected to be included in the present study according to the eligibility criteria.

The main characteristics of the 23 observational studies selected for inclusion in the systematic review are presented in Table 1. A total of 18 studies were conducted on the adult general population, and 5 in individuals with health conditions. Nineteen had a cross-sectional design, while four [14,15,21,24] studies provided longitudinal data on exposure or outcome, yet were cross-sectionally analyzed. Eighteen reported on both sexes, 2 solely on males and 3 on females. Concerning the geographic area, 16 articles’ studies were conducted in Europe, 5 in North America and 2 in Asia, with the majority of the studies being conducted in the Mediterranean basin (*n* = 13). Most studies applied subjective scales and tools as well as clinical symptoms to assess sleep quality features. Dietary intakes were most often registered by food frequency questionnaires (FFQs) and adherence to the Mediterranean diet assessed through a variety of instruments as listed in Table 1.

### 3.2. Mediterranean Diet and Sleep in Mediterranean Countries

Among investigations conducted in the Mediterranean region, a longitudinal study conducted on 1596 participants (aged ≥ 60 years) reported that high adherence to the Mediterranean diet was associated with a lower risk of changes in sleep duration and a better sleep quality [odds ratio (OR): 0.44, 95% confidence interval (CI): 0.29, 0.68, *p* < 0.001)] [14]. However, the majority of studies published to date had a cross-sectional design. A study including 172 Italian participants (mean age 52 years) to the obesity-prevention program the Obesity, Programs of Nutrition, Education, Research and Assessment of the best treatment (OPERA) investigated the influence of adherence to Mediterranean diet and sleep quality, assessed with the Pittsburgh Sleep Quality Index (PSQI); the authors showed that participants with lower PSQI scores (“good sleepers’’) had higher adherence to the Mediterranean diet compared to “poor sleepers”, who reported higher PSQI scores (*p* < 0.001) [22]. In another cross-sectional study conducted among 1936 Italian adults from the Mediterranean healthy Eating, Aging, and Lifestyles (MEAL) study, recruited among the general population in Southern Italy, reported a higher prevalence of adequate sleep quality among individuals with higher adherence to the Mediterranean diet (Q1: 72.4% vs. Q4: 58.9%; *p* < 0.001) and increased likelihood of better sleep quality compared to those less adherent (for the highest vs. lowest quartile, OR = 1.82, 95% CI: 1.32, 2.52; 1-point increase, OR = 1.10, 95% CI: 1.05, 1.16) [17]. When considering weight status, a significant association between adherence to the MD and sleep quality was found in normal/overweight individuals (for the highest vs. lowest quartile, OR = 2.30, 95% CI: 1.49, 3.54; 1-point increase, OR = 1.10, 95% CI: 1.04, 1.16), but not in obese participants [17]. Similarly, in a cross-sectional study, involving 1639 subjects (mean age of 73 years) from the Hellenic Longitudinal Investigation of Aging and Diet (HELIAD), Mediterranean diet adherence was positively associated with sleep quality (*p* < 0.001) and in particular in participants aged ≤ 75 years, whereas there was no association with sleep duration [16]. From the same cohort, another study investigating the probability of prodromal symptoms of Parkinson’s disease and Mediterranean diet adherence showed that daytime somnolence was less prevalent in the higher quartile of Mediterranean diet adherence compared to the lower (43.2% vs. 49.6%, respectively; *p* = 0.029) [18]. Furthermore, a cross-sectional study, involving 957 subjects (median age 50 y) enrolled among indigenous and minority populations in Northeastern Greece showed an association between high Mediterranean diet adherence and long sleep duration (*p* = 0.002), long time in bed (*p* = 0.002) and better sleep quality (*p* = 0.009) [28]. Finally, a French multicenter population study involving 5886 subjects aged 65 years and older investigating sociodemographic, behavioral, and clinical parameters associated with insomnia symptoms (difficulty with initiating and maintaining sleep) showed that higher adherence to the Mediterranean diet was inversely associated with having insomnia symptoms, but only in women [13]. In contrast to the aforementioned studies, some investigations reported null findings. A cross-sectional study including 70 individuals (mean age 53 years) enrolled in the context of the FIT-AGEING study, an exercise-based randomized controlled trial conducted in Spain showed no association between Mediterranean diet adherence assessed through the 14-point questionnaire PREDIMED and sleep quality assessed with PSQI and wrist accelerometers [20]. Finally, few studies have been conducted in the context of the COVID-19 lockdown, showing null results. A study on a convenient sample of 745 adults (median age of 39 years) who resided in Cyprus during the Spring 2020 lockdown [25] and an Italian cross-sectional study conducted on 604 individuals (mean age of 29.8 years) [26] aiming to evaluate the effects of COVID-19 lockdown on dietary habits and sleep quality reported significant findings on the association between Mediterranean diet adherence and sleep quality in men, but not in women. It is noteworthy to underline that these studies were conducted under special conditions and were hard to compare with other general-population studies.

### 3.3. Mediterranean Diet and Sleep in Non-Mediterranean Countries

Most significant findings have been reported also in studies conducted in non-Mediterranean countries. In a longitudinal study conducted on two cohorts (Nurses’ Health Study and the Health Professionals Follow-up Study including 11,493 women with a mean age of 42 years and 5907 men with a mean age of 57 years, respectively) the association between the Mediterranean diet adherence and sleep quality (through the Mayo Sleep Questionnaire and The Epworth Sleepiness Scale used to measure excessive daytime sleepiness) considered as a prodromal feature of Parkinson’s disease was tested. While the diet was examined from 1986 every 4 years, sleep features were tested in 2014–15 resulting in an inverse association between high Mediterranean diet adherence and excessive daytime sleepiness [21]. Also, another cohort study involving 402 women with a mean age of 37 years established a significant association between the Mediterranean diet and sleep quality after a 1-year follow-up (β = −0.30 ± 0.10, *p* < 0.01); moreover, they observed a significant association between Mediterranean diet adherence and lower sleep disturbances (β = −0.30 ± 0.12, *p* = 0.01) and higher sleep efficiency (*p* = 0.01) [24].

Among studies focusing on sleep duration, a longitudinal study examining the relation between Mediterranean-style diet, sleep duration and insomnia symptoms in a total of nearly 2000 participants showed that compared with low MD adherence, moderate–high adherence was associated with a healthier sleep time (6–7 vs. <6 h/night; *p* < 0.01) and less insomnia symptoms (*p* < 0.05). Furthermore, participants with an unchanging MD score in the preceding 10 years had fewer insomnia symptoms compared to participants with a decreasing score (*p* < 0.01) [15]. Similarly, a cross-sectional study conducted on 22,627 individuals (mean age of 61 years) from the Swedish EpiHealth cohort study, showed that short sleepers (<6 h/night) were less likely to have high adherence to the Mediterranean diet (OR = 0.70, 95% CI: 0.56, 0.87), also in combination with sleep quality (OR = 0.67, 95% CI: 0.52, 0.86) [23]. Another cross-sectional study conducted on 2169 Costa Rican adults showed a significant association between inconsistent weekday–weekend sleep and lower Mediterranean diet scores that did not change with sex (β: −0.08, CI: −0.16, 0.006; P, interaction with sex = 0.93); in women, compared with the recommended sleep duration, sleep duration <7 h per night was associated with a lower score (β: −0.35, CI: −0.63, −0.07 *p* = 0.016) [29]. Other studies further investigated overall sleep features, including daytime sleepiness, insomnia symptoms, and overall sleep quality. A cross-sectional study involving 917 Arab women (mean age 36) exploring the relationship between Mediterranean diet adherence, sleeping habits, and insomnia showed a significant association between their Mediterranean-diet-adherence score and better sleep and reduced insomnia symptoms (*p* = 0.015) [27]. A cross-sectional study involving 400 health professionals, male adults working in different healthcare centers affiliated to Baqiyatallah University of Medical Science across the Tehran province, investigated the relationship between MIND diet, sleep quality (PSQI), daytime sleepiness (Epworth Sleepiness Scale; ESS) and insomnia (Insomnia Severity Index; ISI): individuals with higher adherence to the MIND diet had 42% lower odds of daytime sleepiness (95% CI: 0.35, 0.98; *p*-trend = 0.044); moreover, the highest tertile of MIND diet adherence was significantly associated with better sleep quality (T3 vs. T1, OR = 0.58, 95% CI: 0.34, 0.98; *p*-trend = 0.042) and lower odds of insomnia in the multivariate-adjusted model (T3 vs. T1, OR = 0.54, 95% CI: 0.31, 0.93; *p*-trend = 0.031) [30]. However, in a cross-sectional study, a total of 970 men (mean age 71 years) from the Uppsala Longitudinal Study of Adult Men (ULSAM) were involved to investigate the potential influence of Mediterranean diet adherence on sleep disturbance; no significant association between adherence to the Mediterranean diet and sleep quality was reported among the participants [19].

### 3.4. Mediterranean Diet and Sleep in Health Conditions

Some studies explored the association between the Mediterranean diet and sleep features in individuals with health conditions. A total of 309 women with breast cancer were involved in a cross-sectional study to investigate the influence of Mediterranean diet adherence on quality of life: in the multivariate model adjusted for age, cancer stage, BMI, type of surgery, comorbidities and combined therapy, a significant inverse association between Mediterranean diet adherence and insomnia was observed (β = −0.131; *p* = 0.029) [32]. A cross-sectional study included 563 patients with multiple sclerosis aged between 18 and 65 years to evaluate the effects of the Mediterranean Diet on sleep disturbance: the results showed a significant inverse association between Mediterranean diet adherence and sleep disturbance (β: −0.187; 95% CI: −0.358, −0.017; *p* = 0.032) [34]. Another cross-sectional study investigated the association between Mediterranean diet adherence and insomnia in 269 individuals with obstructive sleep apnea (OSA) aged between 21 and 70 years. The authors reported that the patients with the highest Mediterranean diet adherence had 53% lower odds of having insomnia (OR = 0.47, 95% CI: 0.23, 0.95) [33]. In contrast, in a cross-sectional study, 162 participants with OSA with a mean age of 67 years were recruited to focus on the potential effects of MIND adherence on sleep quality but results showed no significant association between MIND and sleep problems and daytime sleepiness [35]. Moreover, a cross-sectional study conducted on 205 patients with rheumatoid arthritis with a mean age of 53 years investigated a possible correlation between sleep quality and Mediterranean diet adherence: results showed that after adjustment, no significant association was observed between Mediterranean diet and sleep score [31].

## 4. Discussion

In this study, observational studies investigating the relationship between adoption of the Mediterranean diet and sleep-related outcomes were systematically reviewed. The evidence evaluated in this systematic review investigated the relationship between adherence to the Mediterranean diet and sleep quality, including duration, morning sleepiness, and insomnia. Overall, most studies showed that higher adherence to a Mediterranean dietary pattern was associated with better sleep features. Although most studies had a cross-sectional design, the findings are in line with those reported from randomized clinical trials, suggesting that dietary intervention with a Mediterranean diet may improve sleep features [36,37].

The main characteristics of the Mediterranean diet may affect brain health and, consequently, be related to sleep features [6,38]. As previously mentioned, the Mediterranean diet is mainly a plant-based dietary pattern characterized by richness in antioxidant vitamins (i.e., vitamins C and D) and phytochemicals, such as (poly)phenols, which may contribute to preventing microglia activation, reduction of pro-inflammatory cytokine production, and ultimately inhibit neuroinflammation [39]. At the molecular level, (poly)phenols can inhibit the activation of certain transcription factors, such as the nuclear factor-kappa B (NF-κB), that are known to be involved in the cellular inflammatory response, leading to the downregulation of the production of mediators, including cytokines such as interleukin-1β (IL-1β) and tumor necrosis factor-alpha (TNF-α) [40]. Moreover, (poly)phenols demonstrate neuroprotective effects by modulating signaling pathways within neurons, for instance by activating the nuclear factor erythroid 2-related factor 2 (Nrf2) pathway, involved in the cellular antioxidant activities [40], and stimulating the sirtuin family receptors, which regulate some cellular processes related to inflammation and stress response [40]. Besides phytochemicals, plant richness in minerals, such as zinc and magnesium, has been called out as potentially favoring sleep quality by the promotion of inhibitory effects on the brain and some regulation of sleep and wakefulness [41,42].

Some effects on the central nervous system may also depend on the quality of macronutrients contained in characteristic foods included in the Mediterranean dietary pattern. The Mediterranean diet is, in fact, considered a relatively high-fat, high-carbohydrate diet: however, consumption of fish, olive oil, and oilseeds assure a high consumption of healthy fats, such as mono- and poly-unsaturated fatty acids (MUFA and PUFA, respectively), which have demonstrated anti-inflammatory properties, playing a role in serotonergic and dopaminergic transmissions, and influencing membrane stability, signal transduction, and fluidity [43]. Specifically concerning the anti-inflammatory mechanisms, omega-3 PUFA eicosapentaenoic acid (20:5, *n*-3; EPA) and docosahexaenoic acid (22:6, *n*-3; DHA) are precursors of 3-series prostaglandin (PG), 5-series leukotrienes (LT), and 3-series thromboxane (TX), which are reported to compete with eicosanoids synthesized from omega-6 arachidonic acid (20:4, *n*-6; AA) and exert anti-inflammatory and pro-resolving properties [44]. Moreover, preference for home cooking as opposed to ready-to-eat meals, having fruits and nuts as snacks over unhealthy snacks, and supposedly low consumption of meat products in favor of plant sources of proteins (i.e., legumes) or poultry, eggs and dairy products may lead to lower consumption of hydrogenated trans fats and long-chain saturated fatty acids [45,46]. Both trans-fatty acids and long-chain saturated fatty acids can activate Toll-like receptors (TLRs) on the surface of immune cells, including microglia in the brain, which initiates a signaling cascade that leads to the production of pro-inflammatory cytokines; the pathways activated involve the Nuclear Factor-kappa B (NF-κB) and the Mitogen-Activated Protein Kinase (MAPK) signaling pathways (including extracellular signal-regulated kinase (ERK), c-Jun *n*-terminal kinase (JNK), and p38 MAPK, which can lead to the expression of inflammatory mediators and the activation of transcription factors that promote inflammation in macrophages, monocytes, and monocyte-derived dendritic cells [47]. Concerning carbohydrates, the main source of daily energy in the Mediterranean diet should be provided by whole-grain products: a high dietary content of carbohydrates is generally related to better sleep, while, conversely, lower intake has been associated with a longer time spent in slow-wave sleep and less time in rapid-eye-movement (REM) sleep [48]. Moreover, the high content in complex molecules and fiber would provide a lower glycemic index, which has been related to improved sleep quality [49]. Conversely, a high intake of added simple sugars (supposed to be low in the Mediterranean diet) has been associated with a worsening of hippocampal function possibly mediated by neuroinflammatory processes [50]. The neurophysiological mechanisms behind such observations are still under investigation: one can hypothesize that carbohydrates may affect tryptophan content in the central nervous system, which is needed to synthetize serotonin, also important for melatonin production [51]. Another potential mechanism relies on the hypothesis that glucose-sensing neurons in the hypothalamus may affect hypothalamus [52].

Besides the direct role of macronutrients toward brain function, the content in short-chain saturated fats, fiber, and protein quality (plant vs. animal protein), as well as non-nutrient compounds (i.e., polyphenols), has been demonstrated to modulate the gut microbiota, which in turn may exert certain effects on the brain via the gut–brain axis and potentially affect sleep features [53]. Indigestible complex carbohydrates, when unprocessed by human enzymes, undergo fermentation by the intestinal flora, resulting in the production of short-chain fatty acids (SCFAs) during fermentation [54]. These SCFAs, encompassing acetate, propionate, and butyrate, exhibit anti-inflammatory effects that can extend to the brain through neuronal and glial signaling pathways, as well as immune system activation. An imbalance in intestinal flora, known as “dysbiosis”, may induce heightened permeability of the intestinal mucosa, leading to a condition commonly referred to as “leaky gut” [55]. Consequently, bacterial elements, such as lipopolysaccharides (LPS) derived from bacterial cell walls, attach to circulating macrophages and monocytes, triggering an inflammatory response (activation of the immune system with promoted synthesis of pro-inflammatory cytokines) [56]. Moreover, bioactive compounds such as (poly)phenols have also been shown to modulate the activation of genes involved in circadian clock genes and leptin, the hormone regulating the energy balance by suppressing hunger [57]. Disruptions in the sleep–wake cycle may lead to variations in the taxonomic configurations of gut microbiota, which in turn have been associated stress/anxiety episodes and sleep-related issues [58,59].

The results presented in this systematic review are strengthened by good consistency across studies and the inclusion of some relatively large cohorts. However, the findings should be interpreted in light of some limitations. First, the observational design of the studies included does not allow for assessing the direction of the association or a causal relationship. Several studies provided cross-sectional analyses, further limiting the evidence concerning a possible causative role of diet on sleep or vice versa. Second, adherence to the Mediterranean diet was generally calculated through derivative methods, such as FFQs and 24 h recalls, which are likely to be limited by recall bias and affected by social desirability bias, which in turn could lead to either under or over estimation of food intake. Third, most of the studies evaluated sleep features using only self-reported questionnaires and not objective tools (i.e., actigraphy). Lastly, the large variety of tools used to test sleep features, instruments to evaluate the level of adherence to the Mediterranean diet, and risk measures to assess the associations between these factors made it impossible to perform a quantitative summary analysis of the evidence.

## 5. Conclusions

In summary, adoption of a Mediterranean dietary pattern appears to be linked with improved sleep characteristics. Despite the possibility of a causal relationship, the outcomes of this study consistently demonstrate that higher adherence to the Mediterranean diet is associated with overall better sleep quality. These findings could support the inclusion of the Mediterranean diet in intervention programs to promote healthy eating aiming to improve sleep quality or sleep-related features. The appealing aspect of the Mediterranean diet, which includes flavorful foods with inherently robust organoleptic and nutritional properties, could be an additional incentive for its adoption especially if compared to interventions limiting certain foods’ or nutrients’ intake.

## Figures and Tables

**Table 1:** The main characteristics of the observational reporting on the relationship between Mediterranean diet and sleep features.

Author, Year, Country	Total Population (Analyzed Sample), Sex, Age (Average)	Dietary Assessment Method	Mediterranean-Diet-Adherence Assessment	Outcome	Outcome Assessment Method	Main Findings
*Adult general population*						
Jaussent, 2011, France [13]	5886 FM, >65 y	FFQ	11-item MD score	Insomnia symptoms	Self-reported questionnaire	Higher adherence to the MD was inversely associated with having insomnia symptoms; however, this was only in women.
Campanini, 2017, Spain [14]	1596 FM, 71 y	Diet history	MEDAS	Sleep duration and quality, daytime sleepiness	Self-reported questionnaire, ESS	Higher adherence to the MD diet was associated with better sleep quality and with lower risk of changes in sleep duration at follow-up.
Castro-Diehl, 2018, USA [15]	1927 (for sleep duration) and 1988 (for insomnia symptoms) FM, 69 y	120-item FFQ	aMed score	Sleep duration, insomnia symptoms	Actigraphy, WHIIRS	Individuals with moderate–high adherence to the MD were more likely to have an adequate sleep duration (6–7 vs. <6 h/night; *p* < 0.01), when compared to those with low adherence, and less likely to report insomnia symptoms occurring with short sleep (vs. no insomnia or short sleep alone; *p* < 0.05).
Mamalaki, 2018, Greece [16]	1639 FM, 73 y	69-item semi-quantitative FFQ	MedDietScore	Sleep quality and duration	12-item Medical Outcomes Study Sleep Scale, self-reported sleep duration	Sleep quality was positively associated with MD adherence (*p* < 0.001), whereas no association with sleep duration was found. When taking into account age, sleep quality was positively associated with MD adherence only in participants aged ≤75 years (*p* < 0.001).
Godos, 2019, Italy [17]	1936 FM, ≥18 y	FFQ	MEDI-LITE score	Sleep quality	PSQI	For each point increase of the MD score, individuals were 10% more likely to have adequate sleep quality. When considering weight status, a significant association between adherence to the MD and sleep quality was found in normal/overweight individuals, although not in obese participants.
Maraki, 2019, Greece [18]	1731 FM, 73 y	Semi-quantitative FFQ	MeDi Score	Daytime sleepiness	12-item Medical Outcomes Study Sleep Scale	Daytime sleepiness was less prevalent among individuals with higher adherence to the MD (*p* = 0.029).
van Egmond, 2019, Sweden [19]	970 M, 71 y	7-day food diary	mMDS	Sleep quality	Self-reported questionnaire	No significant association between MD adherence and sleep parameters was found.
Jurado-Fasoli, 2020, Spain [20]	70 FM, 53 y	24-h dietary recall	14-item PREDIMED score	Sleep quality, sleep parameters	PSQI, wrist accelerometers	No correlation between adherence to the MD and sleep quality or other sleep parameters was found.
Molsberry, 2020, USA [21]	11,493 F, ~48 y (NHS); 5907 M, ~47 y (HPFS)	Semi-quantitative FFQs	aMed score	REM sleep behavior disorder, daytime sleepiness	MSQ, ESS	High long-term adherence to the MD was inversely associated with excessive daytime sleepiness. On the contrary, adherence to the MD was not associated with probable REM sleep behavior disorder.
Muscogiuri, 2020, Italy [22]	172 FM, 52 y	NR	14-item PREDIMED score	Sleep quality	PSQI	A significant association between high MD adherence and better sleep quality was found (*p* < 0.001).
Theorell-Haglow, 2020, Sweden [23]	21,339 FM, 61 y	MiniMealQ FFQ	mMED	Sleep quality, sleep duration	Self-reported questionnaire	Short sleepers had lower adherence to the MD diet, when compared to normal sleepers. Considering sleep quality, short sleepers with poor sleep quality had lower odds of having high adherence to the MD, compared to normal sleepers with good sleep quality.
Zuraikat, 2020, USA [24]	432 F, 37 y	FFQ	aMed score	Sleep quality	PSQI	A significant association between higher baseline MD adherence level and better sleep quality (*p* < 0.01), higher sleep efficiency (*p* < 0.001), and fewer sleep disturbances (*p* < 0.01) was found at 1 y of follow-up.
Kolokotroni, 2021, Cyprus [25]	745 FM, 39 y (median)	NR	MEDAS	Sleep quality	PSQI	No significant association was observed between adherence to the MD and sleep quality during the month of lockdown.
Prete, 2021, Italy [26]	604 FM, 30 y	NR	15-item QueMD score	Sleep quality	PSQI	A significant relation between adherence to the MD and sleep quality was found in men, but not in women (*p* < 0.05).
Zaidalkilani, 2021, Jordan [27]	917 F, 36 y	NR	14-item PREDIMED score	Insomnia	AIS	Adherence to the MD was significantly associated with better sleep and reduced insomnia symptoms (*p* = 0.015).
Georgiadi, 2022, Greece [28]	957 FM, 50 y	Questionnaire on dietary habits	MedDietScore	Time in bed, sleep duration, sleep efficiency	Self-reported questionnaire	High adherence to the MD was significantly associated with longer time in bed (*p* = 0.002), longer sleep duration (*p* = 0.002) and better sleep efficiency (*p* = 0.009).
Gupta, 2022, Costa Rica [29]	2169 FM, ~60 y	135-item semi-quantitative FFQ	AMED score	Sleep duration	Self-reported questionnaire	In women, compared with the recommended sleep duration, sleep duration < 7 h per night was associated with lower adherence to the MD (*p* = 0.016). In unstratified models there was a suggestive association between inconsistent weekday–weekend sleep and lower adherence to the MD.
Rostami, 2022, Iran [30]	400 M, 39 y	168-item semi-quantitative FFQ	MIND score	Sleep quality, daytime sleepiness, insomnia	PSQI, ESS, ISI	Higher adherence to the MIND diet was significantly associated with lower odds of having poor sleep quality (*p* = 0.042), lower daytime sleepiness (*p* = 0.044) and lower odds of insomnia (*p* = 0.031).
*Adults with health conditions*						
Ingegnoli, 2020, Italy [31]	205 FM, 53 y (median), rheumatoid arthritis patients	NR	11-item MD score	Sleep quality	RAID	A significant inverse association was found between sleep and adherence to the MD (*p* < 0.05).
Porciello, 2020, Italy [32]	309 F, 52 y, breast cancer patients	NR	14-item PREDIMED score	Insomnia	EORTC QLQ-C30	A significant inverse association was observed between high adherence to the MD and insomnia (*p* = 0.029).
Kechribari, 2022, Greece [33]	269 FM, 49 y (median), OSAS patients	76-item semi-quantitative FFQ	MedDietScale index	Insomnia	AIS	Higher adherence to the MD was associated with lower odds of having insomnia.
Katz Sand, 2023, USA [34]	563 FM, 44 y, multiple sclerosis patients	NR	MEDAS	Sleep disturbance	ISI	MD adherence was inversely associated with sleep disturbance (*p* = 0.032).
Lawrie, 2023, UK [35]	162 FM, 67 y, Parkinson’s disease patients	FFQ	MIND index	Sleep problems, daytime sleepiness	ESS, UPDRS	No significant association was found between the MIND diet and sleep.

Abbreviations: AIS (Athens Insomnia Scale); aMed (Alternate Mediterranean Diet); AMED (Alternative Mediterranean Diet Score); EORTC QLQ-C30 (Questionnaire for Quality of Life Assessment in patients with cancer, version 3.0); ESS (Epworth Sleepiness Scale); F (female); FFQ (food frequency questionnaire); h (hour); HPFS (Health Professionals Follow-up Study); ISI (Insomnia Severity Index); M (male); MD (Mediterranean diet); MEDAS (Mediterranean-diet-adherence screener); mMED (modified Mediterranean diet score); mMDS (modified Mediterranean diet score); MIND (Mediterranean-DASH Diet Intervention for Neurodegenerative Delay); MSQ (Mayo Sleep Questionnaire); NHS (Nurses’ Health Study); NR (not reported); OSAS (Obstructive Sleep Apnea Syndrome); PREDIMED (Prevención con Dieta Mediterránea); PSQI (Pittsburgh Sleep Quality Index); QueMD (Mediterranean Diet 15-item questionnaire); RAID (Rheumatoid Arthritis Impact of Disease); UPDRS (Unified Parkinson’s Disease Rating Scale); y (years); WHIIRS (Women’s Health Insomnia Rating Scale).

## Data Availability

Data sharing is not applicable to this article as no new data were created or analyzed in this study.
